# Ontogeny of Rat Thymic Epithelium Defined by Monoclonal Anticytokeratin Antibodies

**DOI:** 10.1155/1990/16952

**Published:** 1990

**Authors:** Miodrag Čolić, Suzana Jovanović, Milijana Vasiljevski, Aleksandar Dujić

**Affiliations:** 1 Institute for Experimental Medicine Military Medical Academy Belgrade Serbia and Montenegro; 2 Institute for Experimental Medicine Military Medical Academy Crnotravska 17 Belgrade 11000 Serbia

## Abstract

Ontogenetic study on the expression of cytokeratin (CK) polypeptides within particular subsets of rat thymic epithelial cells (TEC) has been performed by a large panel of anti-CK monoclonal antibodies (mAbs) using the streptavidin-biotin immunoperoxidase method. Simultaneous presence of two or more CK subunits in the same TEC has been demonstrated by double immunoflouorescence labeling. The obtained results showed that the expression of CK polypeptides in fetal and neonatal thymus differed from the adult patterns. The main difference was observed in expression of CK10, 18, and 19 polypeptides. During fetal ontogeny, CK10 and 18 are markers for most medullary TEC or a subset of medullary TEC, respectively, whereas CK19 is mainly a pan-TEC marker. In the adult animals, they are localized in the cortical and a subset of medullary TEC (CK18), subcapsular/perivascular and some medullary TEC (CK19), or in a subset of medullary TEC and Hasall’s corpuscles (HC) (CK10). The switch in their expression in the cortex was observed during the first two weeks of postnatal life.


*Developmental Immunology*, 1990, Vol. 1, pp. 67–75
Reprints available directly from the publisher
Photocopying permitted by license only

© 1990 Harwood Academic Publishers GmbH
Printed in the United Kingdom

### Ontogeny of Rat Thymic Epithelium Defined by Monoclonal Anticytokeratin Antibodies

MIODRAG ČOLIĆ*, SUZANA JOVANOVIĆ, MILIJANA VASILJEVSKI, and ALEKSANDAR DUJIĆ

*Institute for Experimental Medicine, Military Medical Academy Belgrade, Yugoslavia*

Ontogenetic study on the expression of cytokeratin (CK) polypeptides within particular subsets of rat thymic epithelial cells (TEC) has been performed by a large panel of anti-CK monoclonal antibodies (mAbs) using the streptavidin-biotin immunoperoxidase method. Simultaneous presence of two or more CK subunits in the same TEC has been demonstrated by double immunoflouorescence labeling. The obtained results showed that the expression of CK polypeptides in fetal and neonatal thymus differed from the adult patterns. The main difference was observed in expression of CK10, 18, and 19 polypeptides. During fetal ontogeny, CK10 and 18 are markers for most medullary TEC or a subset of medullary TEC, respectively, whereas CK19 is mainly a pan-TEC marker. In the adult animals, they are localized in the cortical and a subset of medullary TEC (CK18), subcapsular/perivascular and some medullary TEC (CK19), or in a subset of medullary TEC and Hasall's corpuscles (HC) (CK10). The switch in their expression in the cortex was observed during the first two weeks of postnatal life.

KEYWORDS: Rat thymus, cytokeratins, monoclonal antibodies, ontogeny.

### INTRODUCTION

Thymic epithelial cells (TEC) form a supporting network of the thymus and also play important roles in the generation of T lymphocytes. Close contact of TEC with surrounding thymocytes is an important factor in proliferation, differentiation, education, and selection of thymocytes. Interaction with major histocompatibility complex (MHC) antigens on TEC surface as well as humoral factors that they secrete are essential events in these processes (Gelfand et al., 1980). In spite of this, very little is known about the function of particular TEC subpopulations.

During the last few years, a new approach to this problem has been the generation of monoclonal antibodies (mAbs) specific for individual thymic components. The use of these reagents enabled dissection of the thymic epithelium into phenotypically distinct subcapsular/perivascular, cortical, and medullary zones. In addition, a variety of other TEC compartments, especially in the medulla, has been demonstrated (Ritter and Haynes, 1987; Kampinga et al., 1989).

Cytokeratins (CK) are other important markers of the thymic epithelium. MAbs specific for CK polypeptides or CK families revealed a considerable TEC heterogeneity in humans (Laster et al., 1986), mice (Savino and Dardenne, 1988a), guinea pigs (Nicolas et al., 1986), and rats (Čolić et al., 1988a). In our recent work (Čolić et al., 1989), using multimarker analysis of the rat TEC by a panel of mAbs recognizing individual CK polypeptides or pairs, we identified six different TEC subsets, each characterized by a different CK map. In this work, these antibodies have been applied in order to study CK expression in the rat thymus during ontogeny. The obtained data showed their different binding patterns in fetal, neonatal, and adult thymus.

*Corresponding author. Present address: Institute for Experimental Medicine, Military Medical Academy, Crnotravska 17, 11000 Belgrade, Yugoslavia.

TABLE 1
Characteristics and Specifities of Anti-CK mAbs

| MAbs | Isotype | Recognized CK polypeptides[a] | Dilution | Manufacturer | References |
|---|---|---|---|---|---|
| RPN. 1166 (CK8) | IgG1 | 8 | 1:5 | Amersham | Lane, 1982 |
| K.8.13 | IgG2a | 1, 5–8, 10, 11, 18 | 1:50 | ICN | Lazarides, 1982. |
| RPN. 1160 (CK18) | IgG2a | 18 | 1:5 | Amersham | Lane, 1982 |
| RPN. 1165 (CK19) | IgG2b | 19 | 1:5 | Amersham | Lane, 1982 |
| RPN. 1163 (CK type I) | IgG1 | acidic CK | 1:5 | Amersham | Lane, 1982 |
| KL1 | IgG1 | 3, 10 | 1:20 | Serotec | Viac and Brochier, 1983 |
| RPN. 1162 (CK7) | IgG1 | 7 | 1:5 | Amersham | Tolle et al., 1985 |
| K.8.12 | IgG1 | 13, 16 | 1:50 | ICN | Lazarides, 1982 |
| AE5 | IgG | 3 | 1:50 | ICN | Cooper et al., 1985 |
| KS.13.1 | IgG1 | 13, 14[b], 17[b] | 1:50 | ICN | [c] |

[a]Numbered according to Moll's classification (Moll et al., 1982).
[b] Weak immunoreactivity.
[c]Immunoreactivity given by the manufacturer.

TABLE 2
Ontogenetic Analysis of the Expression of CK Polypeptides in the Rat Thymus[a]

| MAbs | TEC subsets | Fetal 15 d | Fetal 17 d | Fetal 19 d | Adult 0 d | Adult 7 d | Adult 14 d | Adult 6 wk |
|---|---|---|---|---|---|---|---|---|
| CK8 | SC/PV | NP | NP | NP | +(p) | +(p) | +(p) | + |
| | C | + | + | + | + | + | + | + |
| | M | + | + | + | + | + | + | + |
| | HC | NP | NP | NP | + | + | + | + |
| CK18 | SC/PV | NP | NP | NP | – | – | – | – |
| | C | – | – | – | +(c) | +(c) | +(b) | + |
| | M | +(f) | +(f) | +(f) | +(c) | +(c) | +(c) | +(c) |
| | HC | NP | NP | NP | – | – | – | – |
| CK19 | SC/PV | NP | NP | NP | +(p) | +(p) | +(p) | + |
| | C | + | + | + | +(a) | +(b) | +(c) | – |
| | M | + | + | + | +(a) | +(a) | +(b) | +(b) |
| | HC | NP | NP | NP | – | – | – | –[b] |
| CKI | SC/PV | NP | NP | NP | +(p) | +(p) | +(p) | + |
| | C | +(b) | +(b) | +(b) | +(a) | +(b) | +(c) | – |
| | M | +(a) | +(a) | +(a) | +(b) | +(b) | +(b) | +(b) |
| | HC | NP | NP | NP | – | – | – | – |
| CK7 | SC/PV | NP | NP | NP | +(f) | +(p) | +(p) | +(p) |
| | C | – | – | – | – | – | – | – |
| | M | – | – | +(f) | +(f) | +(f) | +(c) | +(c) |
| | HC | NP | NP | NP | – | – | – | –(f,p) |
| KL1 | SC/PV | | NP | NP | +(f) | +(f) | +(f) | – |
| | C | | – | +(f) | +(a) | +(c) | +(c) | – |
| | M | NT | +(a) | +(a) | +(a) | +(b) | +(b) | +(c) |
| | HC | | NP | NP | + | + | + | + |
| K8.12 | SC/PV | | NP | NP | – | +(f) | +(f) | +(p) |
| | C | | – | – | – | – | – | – |
| | M | NT | +(f) | +(f) | +(f) | +(c) | +(c) | +(c) |
| | HC | | NP | NP | – | – | – | – |
| KS13.1 | SC/PV | | NP | – | – | – | – | – |
| | C | | – | – | – | +(c)[c] | – | – |
| | M | NT | +(f)[c] | +(f)[c] | +(c)[c] | +(c)[c] | – | – |
| | HC | | NP | NP | – | – | – | – |

[a]SC = subcapsule; PV = perivascular area; C = cortex; M = medulla; HC = Hassall's corpuscles; NT = nontested; NP = nonpresent; a = approx. 75–90% cells positive; b = approx. 25–75% cells positive; c = approx. 10–25% cells positive; p = patches of positive cells; f = few cells positive; fp = few patches on some sections positive.
[b]Peripheral layer of some HC positive.
[c]Weak positivity.

### RESULTS

### Ontogeny of CK Expression within Rat Thymic Epithelium

The use of anti-CK mAbs (Table 1) demonstrated different expressions of particular CK polypeptides in the rat TEC during ontogeny. Table 2 summarizes their main binding patterns.

CK8, which is a pan-TEC marker in adult (6-week-old) animals is also a pan-TEC marker during fetal ontogeny. By analyzing four main phenotypically different zones (subcapsule/perivascular area, cortex, medulla, and Hassall's corpuscles [HC]), it can be seen that the development of subcapsular epithelium occurs in the first two postnatal weeks. In addition, clusters of medullary TEC, identified as small, atypic HC, were first observed in neonatal thymus. The same staining patterns were seen with K 8.13 mAb. During fetal ontogeny, CK18 bound to few cells in the medulla, whereas in the postnatal period, it was also gradually expressed in the cortex (Figs. 1A and 1B). Quite different staining patterns were observed using CK19 mAb. Namely, this CK subunit was mainly a pan-TEC marker in fetal thymus (Fig. 2A) and a marker for subcapsular/perivascular and some medullary TEC in adult thymus (Fig. 2C). Loss of CK19 in cortical TEC occurred in the first 2 weeks of postnatal life (Fig. 2B). Similar, but less strong, staining patterns were seen with CK-type I mAb, recognizing nonidentified acidic CK components. KL1 mAb, detecting 3/10 CK pair in mouse thymus (Savino and Dardenne, 1988a) and probably only CK10 in the rat thymus (Čolić et al., 1989) bound to most medullary TEC in fetal thymus (Fig. 3A) as well as to a subset of medullary TEC and HC in adult rats (Fig. 3C). However, variable patches of cortical epithelium, closer to the corticomedullary region, were KL1+ in the neonatal (Fig. 3b) and early postnatal periods.

CK7 and K.8.12 mAbs showed similar immunoreactivity both in adult and neonatal thymus. However, a number of positive TEC in the medulla and subcapsular/perivascular area gradually increased after birth. AE 5 mAbs detecting CK3 was nonreactive (not shown in Table 2), whereas some weak positivity with KS 13.1 mAb was seen from day 17 of fetal life till day 7 of the postnatal period.

### CK-Defined TEC Subsets in Neonatal Thymus Using Double Immunofluorescence Labelling

The next step in our study was to precisely define CK phenotypes of neonatal TEC and to compare them with the adult patterns, which we previously published in detail (Čolić et al., 1989). To do this, we performed various combinations of double immunofluorescence staining with anti-CK mAbs mutually differing in isotipe specificity.

The first combination, using CK18 and 19 mAbs, showed three different staining patterns. The subset of CK18+ medullary TEC was CK19+, although single CK19+ cells were more numerous (Figs. 4A and 4B). In the cortex, distinct areas of single and double positive cells were observed. Patches of CK19+ subcapsular/perivascular TEC were CK18−. A similar staining pattern was seen using a dual combination of CK18 and KL1 mAbs (not shown).

Double immunostaining with CK8 and CK19 mAbs showed that CK8+ cells were predominantly CK19+, except for some patches in the cortex and a small subset in the medulla, which were CK8+19− (Figs. 5A and 5B).

Using a combination of CK19 and KL1 mAbs, we found that cortical TEC were mainly double positive, whereas in the medulla, three distinct TEC subsets were identified: CK19+KL1+, CK19+KL1−, and CK19−KL1+ (Figs. 6A and 6B). Testing CK18/KL1 mAbs pooled together or CK18/CK19 mAbs as a first step as well as CK19 and KL1, respectively as a second step, we identified single CK19+ (not shown) or KL1+ cells in the medulla (Figs. 7A and 7B).

Finally, to check whether CK19+ and KL1+ TEC comprised the whole medullary epithelial compartment, we performed double staining using CK19 and KL1 mAbs pooled together in combination with K8.13 mAb. A discrete TEC subset positive only with K8.13 mAbs was seen (Figs. 8A and 8B).

Based on the results obtained in this study, we summarized phenotypes of TEC subsets in neonatal thymus (Table 3) and compared them with the adult ones determined previously (Čolić et al., 1989). Table 3 and Fig. 9 show the differences in CK polypeptides between neonatal and adult thymus in all phenotypic zones. The main difference was observed in the cortex. In the medulla of the neonatal thymus, we did not identify the CK8+10−18+19+ subset present in adult thymus.

In addition, in the neonatal thymus, the percentage of CK8+18+19+10+ cells was higher, whereas the percentages of CK8+18−19−10− was smaller compared to the adult patterns.

### DISCUSSION

In this study, we applied a large panel of anti-CK mAbs and demonstrated that within rat thymic epithelium, CK are differently expressed in fetal, neonatal, and adult thymus. Various combinations of single and double immunostaining enabled precise characterization of individual TEC subpopulations.

In our previous work (Čolić et al., 1989), we identified six different TEC subsets in adult rat thymus, each characterized by different CK polypeptide expression: four separate medullary TEC, one medullary TEC phenotypically common with subcapsular/perivascular TEC, and one cortical TEC (see also Table 3 in this work). This study extended the previous one and throws new light on TEC ontogeny.

The most marked difference between fetal and adult rat thymus was seen in the cortex. In adult animals, cells in the cortex expressed CK8 and 18, a CK pair very frequently found in simple epithelia

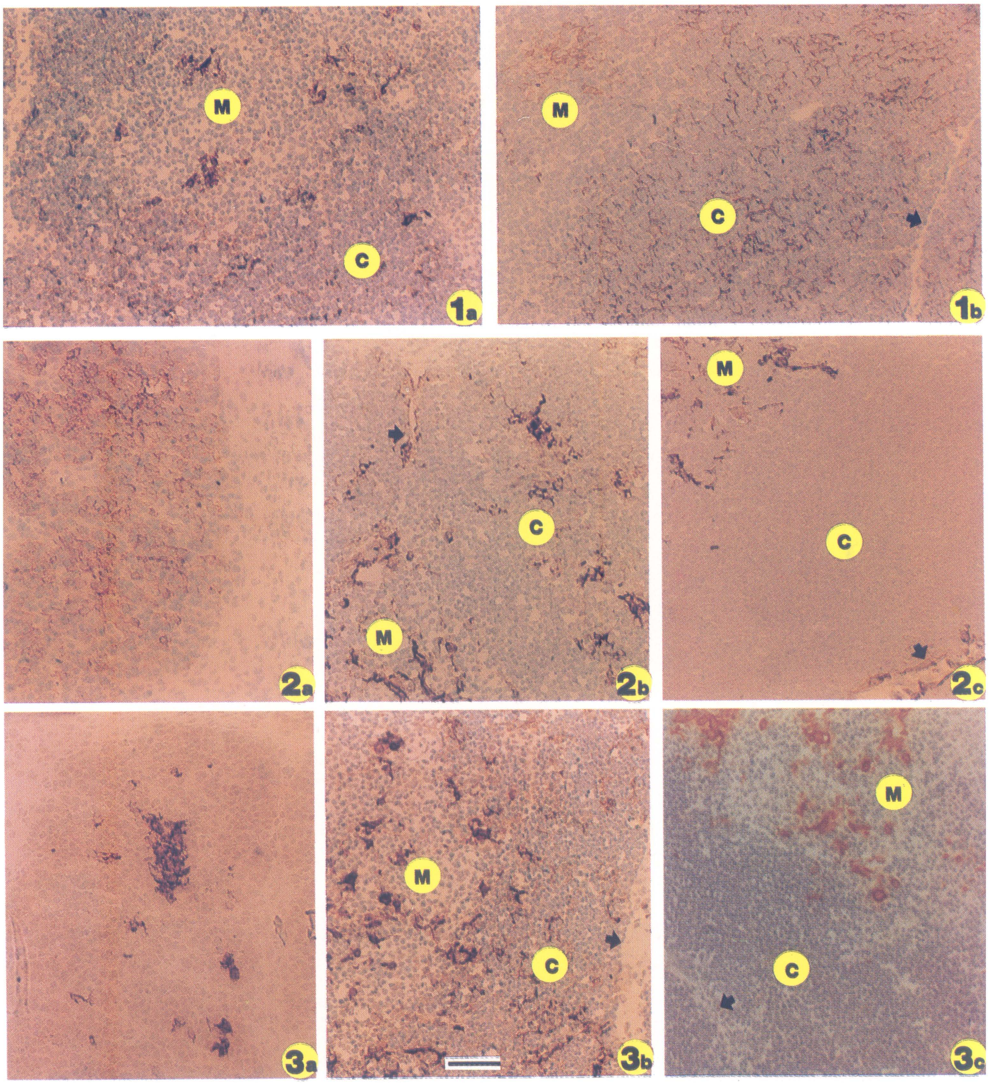

FIGURES 1–3. **Streptavidin-biotin immunoperoxidase staining of the rat thymus with anti-CK mAbs:** 1.(a) Neonatal and (b) adult thymus stained with CK18 mAb. Note a subset of positive cells in the medulla (M) in both figures. Patches of positive cells in the cortex (C) of neonatal thymus or almost all cells in adult thymus are seen, while the subcapsule (arrow) is negative. 2.(a) Fetal (17 days), (b) neonatal, and (c) adult thymus stained with CK19 mAb. Note mainly panepithelial staining in fetal thymus. Most medullary TEC (M), patches of cortical TEC (C), and a part of subcapsular/subtrabecular TEC (arrow) are positive in neonatal thymus. A subset of medullary TEC (M) and subcapsular TEC (arrow) are positive in adult thymus. Cortical TEC (C) are negative. 3.(a) Fetal (17 days), (b) neonatal, and (c) adult thymus stained with KL1 mAb. Substantial numbers of medullary TEC are stained in fetal thymus. Most medullary TEC (M) and patches of cortical TEC (C) are positive in neonatal thymus, whereas the subcapsule (arrow) is negative. A subset of medullary TEC (M) is positive in adult thymus, and no staining in the cortex (C) and subcapsule (arrow) is seen. Bar for all figures = 80 μm. (See Colour Plate I at the back of this publication.)

(Cooper et al., 1985), whereas in fetal life, CK8 and 19 were present. Cortical TEC, both in fetal and adult mice, possess the same CK8/18 polypeptides (Savino and Dardenne, 1988a).

CK19 is expressed in glandular tissues, independent of their origin and in some nonkeratinized stratified epithelia (Quinlan et al., 1985). Although this CK is mainly a pan-TEC marker during fetal ontogeny, in adult rat thymus, its expression is restricted to subcapsular/perivascular epithelium and to a subset of medullary cells. In addition, we previously demonstrated rare CK19+ cysts in the rat thymus (Čolić et al., 1988a). Subsets of medullary TEC, both in adult and fetal mice positive with CK19, were described by Savino and Dardenne (1988a).

Interestingly, during the first two postnatal weeks in rats, simultaneous loss of CK19 and the expression of CK19 occurred in the cortex. We did not find similar data in the literature, but partial or complete loss of the epitopes recognized by the anti-CK19 mAb was detected in some thymoma (Cooper et al., 1985; Savino and Dardenne, 1988b). The epitopes are probably masked in situ and cannot be detected in tissue sections, but immunoblot analysis revealed their presence under denaturing conditions (Cooper et al., 1985; Savino and Dardenne, 1988b). We found that reexpression of CK19 in a patchy pattern in

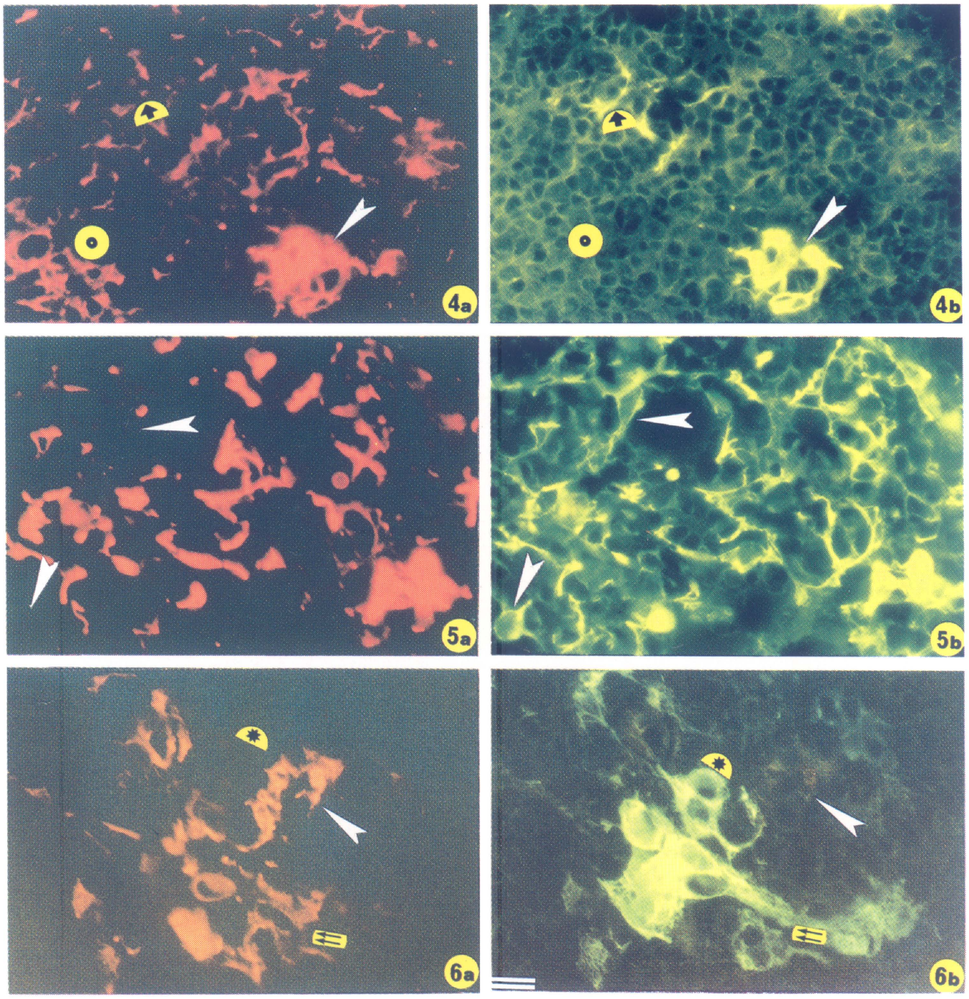

FIGURES 4–6. Double immunofluorescence staining of neonatal rat thymus with anti-CK mAbs: 4.(a) CK19 visualized with TR, and (b) CK18 visualized with FITC. In the medulla, a cluster of double positive cells is indicated by large arrows. In addition, more single positive cells (small circle) is present. In the cortex, patches of CK18+19 weakly+cells (small arrows) and single CK19+ cells are seen. 5.(a) CK19 visualized with TR, and (b) CK8 visualized with FITC. Note the double positive and single CK8+ cells (arrows) in the medulla. 6.(a) CK19 visualized with TR, and (b) KL1 visualized with FITC. Some double positive cells (double arrows), single CK19+ cells (large arrows), and single KL1+ cells (astericks) in the medulla are indicated. Bar for all figures = 20 $\mu$m. (See Colour Plate II at the back of this publication.)

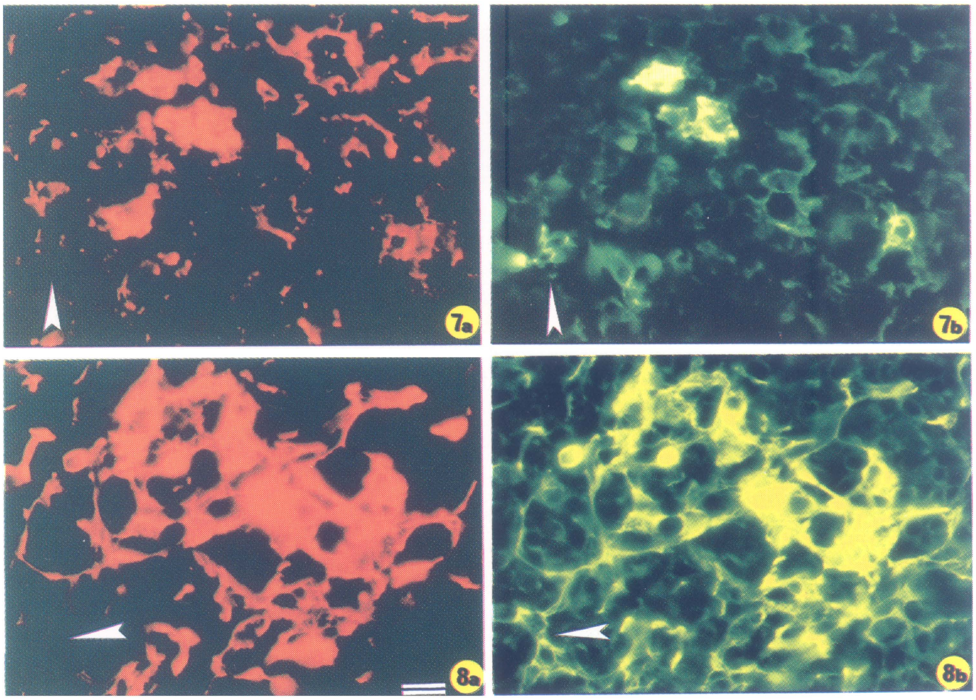

FIGURES 7 and 8. Double immunofluorescence staining of neonatal thymus with anti-CK mAbs: 7.(a) CK18 and 19 mAbs pooled together and visualized with TR, and (b) KL1 mAb visualized with FITC. Single KL1 + cells in the medulla are arrowed. 8.(a) CK19 and KL1 mAbs pooled together and visualized with TR, and (b) K.8.13 mAb visualized with FITC. Single K.8.13 + cells in the medulla are arrowed. Bar for all figures = 20 $\mu$m. (See Colour Plate III at the back of this publication.)

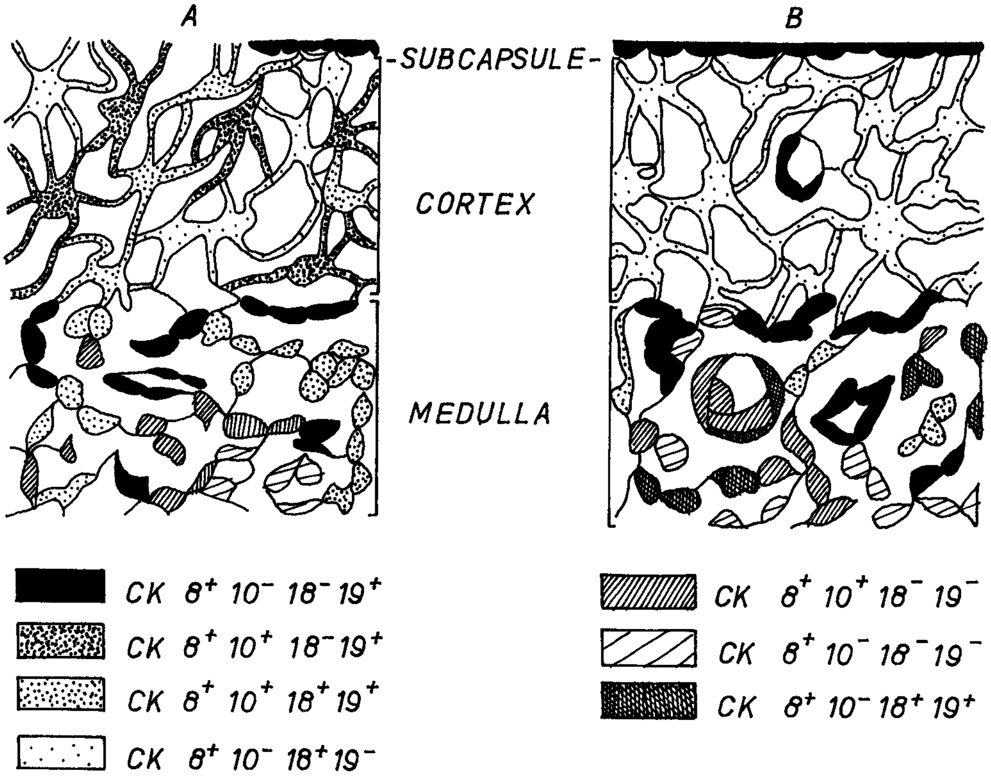

FIGURE 9. Shematic representation of CK-defined TEC subsets in (A) neonatal and (B) adult rat thymus.

outer thymic cortex of adult rats recovering after sublethal X-ray irradiation (nonpublished observations). It is not clear what is the significance of this phenomenon.

TABLE 3
Cytokeratin-Defined TEC Subsets[a] in Neonatal and Adult Rat Thymus

| Localization | TEC subsets | |
|---|---|---|
| | Neonatal thymus | Adult thymus |
| Subcapsule/ perivascular area | CK8 +10 −18 −19 +[b] | CK8 +10 −18 −19 + |
| Cortex | CK8 +10 +18 −19 +<br>CK8 +10 −18 +19 −<br>CK8 +10 +18 +19 + | CK8 +10 −18 +19 − |
| Medulla | CK8 +10 +18 +19 +<br>CK8 +10 −18 −19 +<br>CK8 +10 +18 −19 −<br>CK8 +10 −18 −19 − | CK8 +10 +18 +19 +<br>CK8 +10 −18 −19 +<br>CK8 +10 +18 −19 −<br>CK8 +10 −18 −19 −<br>CK8 +10 −18 +19 + |

[a]TEC subsets in adult thymus are determined previously (Čolić et al., 1989).
[b]Only patches of TEC are positive (staining with CK10 in this subset is given as negative although some rare cells express CK10 polypeptide).

More complex CK organization was found in the medulla, both in neonatal and adult thymus. Most medullary TEC share common CK polypeptides either with subcapsular/perivascular TEC or with cortical TEC. CK8/KL1+ cells or cells positive only with CK8 are two separate TEC subsets found only in the medulla. KL1 mAb recognizing 3/10 CK pair in mice (Savino and Dardenne, 1988a) probably detects CK10 in rats, because we did not identify any positive cells with AE5 mAb specific for CK3. KL1 recognizes only CK10 in human thymus, too (Haftek et al., 1986). The positivity of HC and a subset medullary TEC with KL1 mAb in adult rat (Čolić et al., 1989; this work) and mouse (Nicolas et al., 1985) or HC in guinea pig (Nicolas et al., 1986) indicates that CK10 is a marker for terminally differentiated thymic epithelium. Our data presented here clearly show that this CK subunit has a different distribution during ontogeny. Namely, in the early postnatal period, CK10 is expressed in most medullary TEC and also in a patchy pattern in the cortex, predominantly together with CK19 polypeptide. However, single CK10+ cells were present as early as 17 days of fetal life (not shown) and their numbers increased with age. It is not clear whether these cells originate from a common CK10+19+ subpopulation or not.

Subcapsular/perivascular TEC share common CK polypeptides 7, 8, 16, and 19 with a subset of medullary TEC, indicating their common origin. A panel of monoclonal antibodies with the same specificity has been produced in the human thymus (reviewed by Ritter and Haynes, 1987) or in the rat thymus (Čolić et al., 1988b; Kampinga et al., 1989). Our data showed that the subcapsular epithelium in the rat thymus started to develop after birth. The patches of positive cells were first seen in a deep paraseptal area, sometimes close to the central medullary zone. The appearance of this cell layer in fetal human thymus was seen earlier, at 12 weeks of gestation (Lobach and Haynes, 1987). Our results are in accordance with those published by Crouse et al. (1985). They described a mechanism in which an outgrowth of epithelial cells from a central core in the thymic primordium (branchial cleft) surrounds the outer branchial pouch and forms the subcapsular zone.

The main question resulting from this study is the interrelationship of the rat TEC in terms of its origin. It is largely accepted that in mammals, thymic epithelium is derived from both endoderm (third pharyngeal pouche) and ectoderm (third pharyngeal cleft) (reviewed by Lampert and Ritter, 1988). Close physical contact between them and probably their mutual inductive signals are necessary for normal thymic development. However, controversy over the exact contribution of each compartment in development of TEC compartments has been described.

First studies indicated that medullary epithelium, due to its central localization and the persistence in nude mice mainly in forms of tubules and cysts, was endodermal, whereas the cortex was ectodermal in origin (Cordier and Heremans, 1975). Recent comparative investigations using a large panel of anti-TEC mAbs showed that subcapsular and medullary epithelium shared many molecules with epidermal cells, indicating common ectodermal origin of the skin and thymic medulla (Ritter and Haynes, 1987). More recently, Lampert and Ritter (1988) suggested that thymic epithelium could be derived from a common epithelial stem cell. The main findings supporting this hypothesis are thymic tumors (Willcox et al., 1987) that simultaneously express both cortical and medullary TEC markers. Ontogenetic studies in mice also favor this hypothesis (Lampert and Ritter, 1988).

If we compare our ontogenetic study on CK expression in the cortex and medulla of the rat thymus, we cannot find any strong evidence of their dual origins. CK18 is the best candidate as a marker for endodermaly derived thymic epithelium. Our recent work (Mitrovic et al., 1989) showed that in rats, this CK subunit was present in the simple epithelia, predominantly endodermal in origin. No epithelia originating from the ectoderm were positive. However CK18 is not the only marker for cortical TEC. In addition, its distinct ontogenetic expression and the presence of "adult medullary CK types" (CK10 and 19) in the cortex during the fetal and early postnatal periods are not in accordance with this hypothesis. Small subsets of medullary TEC and subcapsular/perivascular TEC that have CK7 and 16 in all ontogenetic period as well as medullary CK18 negative cells could be the candidates for ectodermaly derived epithelium. But the problem is how to explain the origins of other cell types. The simultaneous expression of various common CK polypeptides during ontogeny favors, although does not strongly prove, the hypothesis of a common origin of thymic epithelium. Medullary cells having common and constant CK polypeptides 8, 10, 18, and 19 in all ontogenetic periods could be a common, maybe undifferentiated, TEC subset. Morphologically undifferentiated epithelial cells have been previously described in the corticomedullary region and medulla (von Gaudecher et al., 1986).

In conclusion, this work throws new light on the development of thymic epithelium and raises many questions about the functional significance of so extremely different CK polypeptide expressions in the thymus.

### MATERIALS AND METHODS

### Animals

Thymuses were obtained from A0 rats, bred at the Farm for Experimental Animals, Military Medical Academy, Belgrade, of the following age groups: days 15, 17, and 19 of fetal life, day of birth, days 7 and 14 or 6 weeks of postnatal life. Day 0 of pregnancy was determined by regular controls of the estral cycle and findings of spermatozoides in vaginal smears. Samples were collected from 3–5 animals per time point.

### Antibodies and Reagents

A panel of 10 mouse, anti-CK monoclonal antibodies was used. Their specificity, isotype, dilution, and origin were given in Table 1. Secondary antibodies and reagents such as goat antimouse IgG subclass specific biotinylated antibodies and streptavidin coupled with perioxidase or Texas red (TR) were purchased from Amersham International, United Kingdom. Sheep antimouse IgG subclass specific antibodies conjugated with fluorescein isothiocynate (FITC) were obtained from Serotec, United Kingdom.

### Immunohistochemistry

For immunohistochemistry, two methods were used: streptavidin-biotin immunoperoxidase staining and double immunofluorescence staining.

Streptavidin-biotin immunoperoxidase staining was performed as follows: thymic cryostat sections (5 μm), which were fixed in acetone for 10 min, were first incubated with mAbs for a minimum of 60 min, washed in TBS, pH 7.6 for 10 min, followed by incubation with secondary biotinylated antibodies (IgG subclass specific) diluted 1:100 in TBS for 30 min. After washing in TBS, sections were incubated with streptavidin-peroxidase (1:100) for 30 min. Revelation of the peroxidase activity was performed by a 10-min incubation of the sections in 0.06% DAB (Serva, FRG) in 0.01% $H_2O_2$. Finally, slides were lightly counterstained with hematoxylin and mounted in gelatin/glycerol medium.

Double immunofluorescence was performed in order to determine whether two different CK polypeptides were present in the same TEC. To prove this, sections were incubated with mAbs mutually differing in isotype specificity. The reaction was visualized using subclass-specific secondary reagents coupled with different dyes. The staining sequence was as follows: first, mAb, IgG subclass-specific FITC-coupled antibody; second, mAb IgG subclass specific biotinylated antibody, streptavidin-TR. Sometimes an opposite sequence of secondary antibodies was applied. After washing following each incubation, sections were mounted in glycerol and observed for green and red fluorescence under a fluorescence microscope (Univar III). Specificity of labeling was checked using appropriate controls, including single staining, omitting the first or second mAb, as well as their replacement with an irrelevant mouse mAb of the same isotype (produced in our laboratory). All controls gave negative results.

*(Received August 20, 1989)*

*(Accepted September 26, 1989)*

### REFERENCES

Čolić M., Matanović D., Hegadiš Lj., and Dujić A. (1988a). Heterogeneity of rat thymic epithelium by monoclonal anti-keratin antibodies. Thymus **12:** 123.

Čolić M., Matanović D., Hegediš Lj., and Dujić A. (1988b). Immunohistochemical characterization of rat thymic non-lymphoid cells—I. Epithelial and mesenchymal components defined by monoclonal antibodies. Immunology **65:** 277.

Čolić M., Jovanović S., Mitrović S., and Dujić A. (1989). Immunohistochemical identification of six cytokeratin-defined subsets of the rat thymic epithelial cells. Thymus **13:** 175.

Cooper D., Schermer A., and Sun T.T. (1985). Classification of human epithelia and their neoplasms using monoclonal antibodies to keratins: Strategies, applications, and limitations. Lab. Invest. **52:** 243.

Cordier A.C., and Heremans J.E. (1975). Nude mouse embryo: Ectodermal nature of the primordial thymic defect. Scand. J. Immunol. **4:** 193.

Crouse D.A., Turpen J.B., and Sharp J.G. (1985). Thymic non-lymphoid cells. Surv. Immunol. Res. **4:** 120.

Gaudecker B. Von. (1986). The development of the human thymus microenvironments. *In:* The human thymus: Histophysiology and pathology, Muller-Hermelink H.K., Ed. (Berlin: Springer Verlag), p. 1.

Gelfand E.W., Dosch H.M., Shore A., Limbatibul S., and Lee J.W.W. (1980). Role of thymus in human T cells differentiation. *In:* Biological basis of immunodeficiency, Gelfand E.W., and Dosch H.M., Eds. (New York: Raven Press), p. 39.

Haftek M., Staquet M.J., Viac J., Schmitt D., and Thivolet J. (1986). Immunogold labeling of keratin filaments in normal human epidermal cells with two anti-keratin monoclonal antibodies. J. Histochem. Cytochem. **34:** 613.

Kampinga J., Berger S., Boyd R., Brekelmans P., Čolić M., Van Ewijk W., Kendall M., Ladyman H., Nieuwenhuis P., Ritter M., Schuurman H-J., and Tournefier A. (1989). Thymic epithelial antibodies: Immunohistological analysis and introduction of CTES nomenclature. Thymus **13:** 165.

Lampert I., and Ritter M.A. (1988). The origin of the diverse epithelial cells of the thymus: Is there a common stem cell? *In:* Thymus update, vol. 1, Kendall M.D., and Ritter M.A., eds. (London: Harwood Academic Publishers), p. 5.

Lane E.B. (1982). Monoclonal antibodies provide specific intramolecular markers for the study of epithelial tonofilament organization. J. Cell Biol. **92:** 665.

Laster A.J., Itoh T., Palker T.J., and Haynes B.F. (1986). The human thymic microenvironment: Thymic epithelium contain specific keratins associated with early and late stages of epidermal keratinocyte maturation. Differentiation. **31:** 67.

Lazarides E. (1982). Intermediate filaments: A chemically heterogeneous, developmentally regulated class of proteins. Annu. Rev. Biochem. **51:** 219.

Lobach D.F., and Haynes B.F. (1987). Ontogeny of the human thymus during fetal development. J. Clin. Immunol. **7:** 81.

Mitrović S., Čolić M., and Popović Lj. (in press). Comparative studies on the expression of SHC-9D8 antigen and cytokeratin 18 in rat epithelial cells. Period. Biol.

Moll R., Franke W.W., and Schiller D.L. (1982). The catalog of human cytokeratins: Patterns of expression in normal epithelia, tumors and cultured cells. Cell **31:** 11.

Nicolas J.F., Reano A., Kaiserlian D., and Thivolet J. (1986). Epithelial cell heterogeneity in the guinea pig thymus: Immunohistochemical characterization of four thymic epithelial subsets defined by monoclonal antikeratin antibodies. Eur. J. Immunol. **16:** 457.

Nicolas J.F., Savino W., Reano A., Viac J., and Dardenne M. (1985). Heterogeneity of thymic epithelial cell (TEC) keratins: Immunohistochemical and biochemical evidence for a subset of highly differentiated TEC in the mouse. J. Histochem. Cytochem. **33:** 687.

Quinlan R.A., Schiller D.L., Hatzfeld M., Achstatter T., Moll R., Jorcano J.L., Magin T.M., and Franke W.W. (1985). Patterns of expression and organization of cytokeratin intermediate filaments. Annu. NY Acad. Sci. **455:** 282.

Ritter M.A., and Haynes B.F. (1987). Summary of thymic epithelium workshop. *In:* Leucocyte typing III. White cell differentiation antigens, McMichael, A., Beverley P., Hagg N., and Horton M. Eds. (Oxford: Oxford University Press), p. 247.

Savino W., and Dardenne M. (1988a). Development studies on expression of monoclonal antibody-defined cytokeratins by thymic epithelial cells from normal and autoimmune mice. J. Histochem. Cytochem. **36:** 1123.

Savino W., and Dardenne M. (1988b). Immunohistochemical studies on a human thymic epithelial subset defined by the anti-cytokeratin 18 monoclonal antibody. Cell Tissue Res. **254:** 255.

Tolle H.G., Weber K., and Osborn M. (1985). Microinjection of monoclonal antibodies specific one intermediate filament protein in cells containing multiple keratins allow insight into the composition of particular 10 nm filaments. Eur. J. Cell Biol. **38:** 234.

Viac J., Reano A., Brochier J., Staquet M.J., Thivolet J. (1983). Reactivity pattern of monoclonal anti-keratin antibody (KL 1). J. Invest. Dermatol. **81:** 351.

Willcox N., Shluep M., Ritter M.A., Schuurman H-J., Newson-Davis J., and Christensson B. (1987). Myasthenic and non-myasthenic thymoma. An expansion of a minor cortical epithelial cell subset? Am. J. Pathol. **127:** 447.

